# The Spas of Homburg

**Published:** 1842-10-01

**Authors:** 


### The Spas of Homburg. By Sir *Alexander Downie*, 1842.

The town of Homburg, the capital of a sovereignty, the revenue of which amounts to the enormous sum of 15,000 pounds per annum, lies at the eastern foot of the Taunus mountains, so fertile in mineral springs, and at the distance of ten miles from Frankfort. The population of the whole sovereignty is about 24,000 souls. The town is built on the slope of a hill, and the mineral springs rise in the valley below. It boasts a large Cursaal, and numerous shady walks for the benefit of the spa drinkers. In respect to climate, Sir A. Downie asserts that no part of the Taunus, including the Baths of Nassau, can exceed Homburg for salubrity. The exhalations from saline springs impregnate the mountain air, rendering it similar to sea-air at Scarbro' or Brighton.

In the Taunus chain of mountains ten mineral waters enjoy great celebrity, and Homburg, though last not least. The following table, from Sir Alexander Downie, will shew the ingredients in each of the three chief springs of Homburg.

ANALYTICAL TABLE OF THE SPRINGS OF HOMBURG.
Quantity 16 Ounces.

| NAMES. | Badquelle. | Elizabethan Brunnen. | Stahlbrunnen. |
|---|---|---|---|
| Temperature .. .. .. | 52° Fah. | 53° Fah. | 53½° Fah. |
| Carbonic Acid Gas .. | 22 Cubic Inches. | 48½ Cub. Inches | 32 Cubic Inches. |
| Muriate of Soda .. .. | 108,392 | 79,154 | 79,864 |
| ———Lime .. .. | 15,285 | 7,756 | 10,667 |
| ———Magnesia .. | 5,904 | 7,787 | 5,329 |
| ———Potash .. | 0,384 | — | 1,076 |
| Sulphate of Soda . .. | - - - - | 0,380 | — |
| ———Lime .. .. | 0,212 | — | — |
| Carbonate of Lime .. | 9,698 | 10,982 | 7,534 |
| ———Magnesia | 2,485 | 2,013 | — |
| ———Iron .. | 0,480 | 0,460 | 0,936 |
| Bromate of Magnesia .. | 0,002 | — | — |
| Pure Silica .. .. .. | 0,164 | 9,315 | 0,314 |
| Free Carbonic Acid Gas | - - - - | 21,486 | 21,265 |
| Author .. .. .. . | Mathias. | Liebig. | Liebig. |

Thus we find that the Badquelle contains no less than 139 grains of solid matters—of which, muriate of soda, or common salt, forms 108 grains in the pint. The first two, Badquelle and Elizabethan, contain nearly half a grain of iron—the Stahlbrunnen nearly a grain. They are all three deficient in the aperient neutral salts—as sulphate of soda or of magnesia.

The Badbrunnen or saline is of a turbid yellowish colour, with a bitterish, salt, or almost styptic taste of the most disagreeable kind. It is only used for baths—the water being raised by pumps and carried in casks to the town. There is but one public bathing establishment, where the common warm, cold, vapour, or shower-bath may be enjoyed.

Near to the Badquelle is the SAUER BRUNNEN, or acidulous spring, very brisk and agreeable to the taste, full of carbonic acid gas, and resembling the celebrated waters of Selters and Fachingen. It is highly valued as a beverage.

Lower down the valley is the Elizabethan Brunnen, whose waters are baled out by a pair of peasant girls. This is strongly impregnated with carbonic acid gas—48 cubic inches to the pint—which covers the bitterish salt taste. The *aprѐs gout* is decidedly chalybeate. It forms a refreshing and even luxurious draught in a hot summer morning. It produces a warm glow in the stomach, with feelings of exhilaration. The time for drinking these waters is the same as at other spas—early in the morning. When the bath is judged necessary, eleven o'clock is a good hour. Plain digestible food—roast or boiled beef—mutton—poultry—game—light pudding, are the order of the day here; but pastry, fruit, vegetables, and high-seasoned dishes are prohibited.

The new spring, or STAHLBRUNNEN, is not disagreeable to the taste, though it is difficult to say whether that of salts, iron, or sulphuretted hydrogen prevails, so intimate is the combination, and so influenced by the carbonic acid gas, with which it is so strongly impregnated. Sir Alex. Downie thinks the water resembles a mixture of Cheltenham, Harrogate, and Tunbridge Springs, with a stream of carbonic acid gas passed through it. The pint contains 126 grains of solid matter, being little less than that of the Elizabethan or Curbrunnen, as it is commonly called. It contains more iron than any chalybeate in Germany except that of Liebenstein. It is a powerful ferro-saline spring; being both aperient and tonic.

In the following observations of Sir A. Downie, we perfectly agree.

"In describing the mode of action of the water of Homburg on the human system, I beg leave to refer the reader to a larger work,* in which I have endeavoured to take a general view of this interesting subject, totally overlooked by the majority of writers on mineral waters, or clothed in such mystical language, as to render their views utterly unintelligible. Who, for instance, can comprehend the doctrine of a *peculiar vital principle* in mineral waters, 'communicating,' says Dr. Peez of Wisbaden, 'to the human body either an attractive faculty more consonant with the medicinal component parts of the water, or acting by itself as a healing power upon the diseased organism?' This is, indeed, as Dr. Johnson remarks, a 'good specimen of German ideality and transcendental mystification.' I may be permitted to add, that all that has been produced by the pen of this *remarkable author* on the subject of mineral waters, and I am not aware that he has ever attempted to publish his peculiar opinions on any other branch of medical science, are involved in what he himself denominates the same 'magic' or mystic 'gloom.' The long and laboured dissertations of the German Spa doctors on crisis and water fever, have also appeared to me more in the light of an attempt to mystify than to illustrate what is in itself both plain and intelligible.

When a mineral water, after being taken for some time, disagrees with the patient, or when, either by the improper use of such water, or it may be some act of imprudence on his own part, a deranged state of the stomach and bowels, with all the concomitant symptoms, is engendered, he is gravely told that he has arrived at the great or little crisis as the case may be—that the water-fever has set in, the mystic change is about to take place, or the *vital principle* in the water has '*stirred up*' his '*internal organism*,' previous to effecting a complete and radical cure of the malady with which he has been afflicted. What, then, it will be readily asked, is the treatment pursued in such cases? Why, to continue the water; the unhappy victim of *spa quackery* is told that if he will only do so, the vital principle already mentioned will attain the mastery, and that relief will be announced by excessive action of the bowels or kidneys—profuse perspiration or violent retching; that is, nature will effect her own relief. The quantity of undigested mineralised matter which has disordered the stomach and bowels, occasioned nausea, headache, and abdominal distention, accompanied by fever and nervous irritability, will be got rid of by vomiting or purging; otherwise, as not unfrequently happens, the foundation of another disease is laid, or the patient dismissed as incurable, or he may be sent on a journey 'to that unknown land whence no traveller returns.'

This, then, is a crisis, or a water-fever, which a few grains of blue-pill and colocynth would have speedily arrested, and thus have enabled the patient to pursue his course without farther inconvenience, if the water really suited his complaints.

During eight years that I have practised at the German spas, I have not seen

"* The Efficacy of Mineral Waters in the Treatment of Chronic Disease, pp. 13—33."

one case of what is called crisis or 'Bad Sturm' (storm). I have frequently known the waters of Wiesbaden and other spas to have been improperly administered. I have also seen many cases in which great constitutional disturbance has been occasioned by imprudence in diet, incautious exposure to cold, and similar accidental causes; but conceiving it my duty in this, as in all other cases, to afford relief to the sufferer as speedily as possible, I have never seen such symptoms as those mentioned by Dr. Richter and others—viz. 'dark-coloured, strongly impregnated urine of a peculiar smell containing sometimes *slimy*, unctuous purulent or sandy particles—extremely copious critical alvine evacuations, which sometimes present a cuticular, or slimy, or bloody, or bilious appearance, or take a pitchy or jelly-like consistency, and occur not unfrequently for whole days together—critical secretions through the skin—perspirations of an entirely specific nature, pungent, and often offensive in odour, clammy, and sometimes breaking out in particular places, but often over the whole body, &c. &c.'

I have no doubt the learned doctor has seen such symptoms, and so should I, if, on being called to a patient suffering as I have described, I had prescribed a decoction of herbs or more mineral water, or waited, like the German faculty, till nature had exerted her energies to throw off the load of extraneous matter which had impeded her functions. I should not then have been surprised to find the breath, urine, and perspiration tainted, or a quantity of morbid matter evacuated from the bowels; and I should have seen a case of *crisis*, and have subjected my patient to the horrors of a *bad sturm!!!* That there is a point beyond which mineral waters, when indicated, ought not to be continued, I readily admit. This, in the other work already alluded to, I have called the point of *saturation*. I find that Dr. Johnson uses the same term, but if there is any merit of originality in its selection, we both may lay claim to it, since his work was published before mine, but had not reached Frankfort till after mine had gone to press. This point is marked by an increase in all the secretions, which being formerly in an unhealthy state, are, by the action of the water and baths, restored to their normal condition. Thus, for instance, if, on commencing the waters, the bowels have been obstinately torpid, the skin dry, the urine scanty, the secretions of the mucous membranes imperfect—when, after the waters have been continued for some time, three, four, or six weeks, we find that the bowels are easily stimulated to action by a small quantity of an aperient water,—the secretion of the skin excited on the least exertion—the kidneys susceptible of small quantities of liquid, and the secretions of the mucous membranes restored,—the patient has attained the point of saturation; caution must be enjoined, and the waters discontinued. I do not mean to insinuate that these signs must be present; sometimes they appear in a modified degree, and in many cases not at all. Occasionally eruptions and hemorrhoids are the result of a course of mineral water, and ought to be regarded as salutary."*

The Badquelle, as we have seen, contains more than four times the quantity of saline as the Soolen Sprudel at Kissengen; it is therefore almost too powerful even as a bath. This spring is chiefly employed externally in affections of the skin, scrofula, and syphilitic eruptions. Sir Alexander Downie recommends the water to be diluted with an equal quantity of plain water, when it proves irritating to the skin or produces boils there.

"The varieties of cutaneous diseases in which these baths may be employed with advantage are prurigo, porrigo, psoriasis or tetter, and impetigo; in all these

* Sir A. Downie, p. 47, 51.

cases the Elizabethan Brunnen ought to be taken internally as an auxiliary, the baths being viewed in the light of a primary remedy.

The great advantage of the saline baths of Homburg arises from their employment in conjunction with the internal use of the other spring, in which case we must view the Elizabethan as the primary and the Bad-Quelle as the secondary agent." 53.

The primary effects of the Homburg waters, like those of Kissengen, are aperient, alterative, and tonic. When not sufficiently aperient, some sulphate of magnesia, or what is better, some Pulna water is added. When more tonicity is desired, the New Spring (which is a stronger chalybeate,) may be mixed with it. The waters of this place act on the kidneys, liver, skin, and on the whole of the secreting apparatus and surfaces. When swallowed they produce (as was before observed,) an agreeable feeling of warmth in the stomach, and a pleasing sense of exhilaration. Three beakers usually open the bowels in the course of an hour, unattended with any inconvenience, and their use, even for weeks, is said not to weaken the intestinal canal, unlike many purely saline springs. The kidneys are excited and the appetite is increased. These waters, as well as those of Kissengen, Marienbad, Schwalbach, &c. bear transportation remarkably well, and lose but little of the properties in the transit.

" Let it not be supposed that in these remarks I in any degree advocate the use of mineral waters in acute disease; they are solely applicable to affections of a chronic nature. No one can be more sensible of the value of medicines pharmaceutically prepared in all diseases partaking of an acute character when judiciously administered than I am; but when the malady merges into the chronic form, the continued use of such drugs must prove detrimental to the constitution. The great desideratum, therefore, is to find a substance which will remove discomfort without injury—a safe but efficient remedy, the continued use of which will gradually allay the effects at the same time that it removes the cause. This, I affirm, we have in the mineral waters of Germany when judiciously employed—whether they be drunk at the fountain or imported fresh from the source." 62.

The Homburg waters are to be taken with caution, and under medical advice. In many disorders they will be found hurtful rather than beneficial. They are counter-indicated in all acute, febrile, or inflammatory affections—in tendencies to fulness about the head—in organic diseases of heart, lungs, or other important viscera—and even in sub-acute inflammation of internal structures.

" *Rheumatism and Gout.*—In the generality of afflictions of this nature, Wiesbaden is decidedly the most efficacious spa in Germany; there are cases, however, in which the internal use of this spa is counter-indicated, and the Elizabethan Brunnen of Homburg may be substituted with advantage.

It is quite a mistake to suppose that when an invalid is ordered to Wiesbaden, it follows as a necessary consequence that he is to drink the water as well as to bathe in it. I have known many disagreeable consequences result from the internal use of these waters in cases where the bath was indicated, and when, if taken alone, or in conjunction with a saline aperient water, it was likely to prove eminently serviceable. This I attribute to the difficulty of digesting the water when much gastric irritability is present, a symptom which frequently accompanies gouty and rheumatic complaints; in all such instances, the Elizabethan

Brunnen of Homburg will be found a valuable auxiliary, inasmuch as it will produce the desirable effects of regulating the bowels, improving the digestion, correcting acidity, and strengthening the intestinal canal, at the same time that the bath, acting on the skin and muscular system, will reduce enlarged and swollen joints, and gradually restore the impaired powers of locomotion." 70.

In retrocedent and misplaced gout, Sir A. D. thinks the Homburg waters are safer than those of Wiesbaden; "Owing to the tendency which the *latter* have to drive the disease back to the place formerly attacked." We would draw a conclusion the reverse of the author's on this point. As debility is frequently the consequence of even great relief from gout and rheumatism at Wiesbaden, a chalybeate is often recommended as the NACH KURE. Sir A. thinks the Homburg waters, as containing tonic with aperient properties, are preferable to Schwalbach.

In hepatic affections, Carlsbad has generally borne off the palm; but our author thinks that had the patients been ordered to Marienbad, Homburg, or Kissengen, waters of a ferro-saline nature, they would have fared better than at the Sprudel.

"Thermal waters are decidedly more difficult of digestion than cold, and when symptoms of gastric dyspepsia accompany affections of the liver, it is evident that any water which will have the effect of superinducing nausea, loss of appetite, heart-burn, and putrescent eructations, cannot be continued with advantage. In torpidity of the liver, whether caused by residence in a tropical climate, intermittent fever, gastro-intestinal inflammation, or abuse of spirituous liquors, I can strongly attest the benefit which may be derived from the judicious employment of the water of Homburg." 75.

DYSPEPSIA is a disease to which the waters of Homburg are well adapted. Female complaints, too, are much benefitted by these waters. The accommodations at Homburg, though not equal to those of Wiesbaden or Baden-Baden, are very comfortable and moderate. An omnibus runs daily from Frankfort, in an hour and a half, to the Spa at the trifling expense of ten-pence. It may be reached in four days from London, with ease.

